# Gonial Angle in Forensic Anthropology to Determine Age and Gender: A Population-Specific Analysis

**DOI:** 10.7759/cureus.63481

**Published:** 2024-06-29

**Authors:** Sakshi Sikaria, Abirami Arthanari, Karthikeyan Ramalingam, Vignesh Ravindran, Lavanya Prathap

**Affiliations:** 1 Department of Forensic Odontology, Saveetha Dental College and Hospitals, Saveetha Institute of Medical and Technical Science, Saveetha University, Chennai, IND; 2 Department of Oral Pathology and Microbiology, Saveetha Dental College and Hospitals, Saveetha Institute of Medical and Technical Science, Saveetha University, Chennai, IND; 3 Department of Pedodontics and Preventive Dentistry, Saveetha Dental College and Hospitals, Saveetha Institute of Medical and Technical Science, Saveetha University, Chennai, IND; 4 Department of Anatomy, Saveetha Dental College and Hospitals, Saveetha Institute of Medical and Technical Science, Saveetha University, Chennai, IND

**Keywords:** forensic dentistry, digital orthopantomogram, accuracy, reliability, mandibular parameter, two different age groups, gonial angle, forensic analysis, gender identification, dental age estimation

## Abstract

Background

The study highlights the gonial angle as a key craniofacial landmark for age and gender determination in forensic cases. It emphasizes population-specific analysis, enhancing precision by recognizing variations between populations. By clarifying the gonial angle's forensic use, the study offers clear guidelines, improving forensic practices. Moreover, the gonial angle and age and gender correlations are thoroughly examined, offering important information on their forensic relevance. The results highlight how crucial population-specific research is to improving the precision and dependability of forensic age and gender estimation techniques, which advances forensic anthropology and supports forensic investigations around the globe.

Aim and objective

The purpose of this study is to assess the accuracy of age and gender estimates using gonial angles. The objectives of this research are to evaluate the precision of age and gender estimates utilizing the gonial angle.

Materials and methods

This present study comprises two groups based on age groups: Group I belongs to 51 to 60 years of age, and Group II belongs to 61 to 70 years of age. Making use of G-Power software (version 3.1.9.4, Düsseldorf, Germany), the sample size was determined. The calculation ensured 95% statistical power at a significance level (alpha error probability) of 0.05. To achieve sufficient statistical power, a total of 1000 samples were included, with a projected required sample size of 92. A total of 1000 samples, consisting of 500 male and 500 female panoramic radiographs, were meticulously selected for the study. The samples picked were within the age range of 51 to 70 years. Orthopantomograms were determined using Planmeca software (Planmeca Romexis®, Version 6.0, USA Inc.). Descriptive statistics, including prediction classification analysis of age and gender, were conducted using SPSS Statistics version 16.0 (SPSS Inc., Released 2007, SPSS for Windows, Version 16.0, Chicago, SPSS Inc.).

Results

According to this study, the mean gonial angle of males aged 51 to 60 years is larger (124.7370 degrees) than that of females (119.6371 degrees). The female group's mean estimates are more accurate, as seen by the smaller standard error (0.20844) compared to the male group's (0.60998). A statistically significant difference in mean gonial angles between the genders is evident, with males having a larger gonial angle (p-value <0.001). In the age range of 61 to 70 years, the mean gonial angle of females is higher (128.4322 degrees) than that of males (124.0529 degrees). In this instance, the male group's standard error is smaller (0.14968) than the female group's (0.30028), indicating more accurate mean estimates. Once more, a statistically significant difference is indicated by a p-value of less than 0.001, with females having a larger gonial angle than males.

Conclusion

Our study revealed that the gonial angle of the mandible can be considered a reliable parameter for gender identification. The study's limitation is its inability to reliably identify gender in the subadult population and in cases of edentulousness. An orthopantomogram is a trustworthy and accurate method for taking the different measurements needed to identify the gender of a particular mandible.

## Introduction

Forensic odontology has become a major component of medicolegal practice, where identification of human remains is critical. The field of forensic odontology has a long history that spans the Roman Empire to the current 21st century and is continually evolving [1]. Forensic odontology uses a range of distinctive techniques to assist with victim identification, some of which have the ability to verify age, gender, and identity. The process of identifying age and sex from living or deceased remains is known as forensic examination [2]. The importance of forensic odontology is well-known and has been demonstrated in the literature by a number of in-person incidents and laboratory studies [3]. In forensic odontology, the remnants, primarily made up of teeth and jawbones, allow for the identification of the individual. Patterns of tooth eruptions can be a valuable source of data used to calculate the victim's chronological age [4]. The four main components of forensic anthropology are sex determination, age estimation, size, and ancestry bearing significant weight [5]. A fundamental aspect of biological and forensic anthropology, sex determination is significant in a number of fields, such as population genetics, archeological studies, and forensic investigations. It involves identifying a person's biological sex based on the skeletal remains that they have. Due to its sexually dimorphic characteristics and comparatively high rate of preservation in forensic and archaeological contexts, the mandible is one of the most significant skeleton elements that is regularly examined to ascertain a person's sex. In forensic practice, it is typical to identify unidentified human remains in cases of skeletonization, mutilation, or putrefaction [6].

Aside from the standard assessment of age and gender, human remains can be identified using various landmarks and measurements of many parameters on the mandible. A collection of morphological traits known as sexual dimorphism serves to distinguish males from females. You can tell a male from a female by looking at different skeletal morphologic traits. Gonial angle and ramus height as sex determinants. One of the trickiest things about forensic dentistry is figuring out a person's age and gender. Mandibular factors can be used to establish an individual's age and gender. The mandibular shape, mental form, and gonial angle are a few anthropometry instruments that are employed in sex determination methods [7]. The gonial angle can also be used to estimate age in extreme situations such as mass disasters, exhumed human remains, murderous mutilations, missing people, and so on [8]. The mandible is one of the primary skeletal features that is used to determine an individual's age since it undergoes distinct morphological changes throughout the course of a person's life. Its importance as a biometric instrument is increased by its innate sexual dimorphism, which serves as the basis for sex determination [9].

The degree of sexual dimorphism innate in the population and the completeness of the remains determine how reliable the gender determination is. Gender can be identified with up to 100% accuracy when the complete adult skeleton is available for study, but in mass disaster situations, where bones are typically found in fragments, the ability to determine a person's sex primarily depends on the sections of the skeleton that are available. The most frequently used skeleton part for determining gender is the skull, which is followed by the pelvis. Given that the mandible is the most dimorphic bone in the skull, it may be crucial to determine sex in situations where the whole skull is missing. The mandible is the biggest and strongest bone in the face. Compared to many other bones, it is more resilient and has remained in better condition due to the existence of a dense layer of compact bone. The size and form of the mandible reflect dimorphism that determines gender determination using the mandibular ramus and gonial angle on an orthopantomogram. Because of its sexually dimorphic characteristics, the mandible is very significant when it comes to skeletal remains that are used to determine gender. In particular, the mandible's gonial and antegonial angles have attracted attention as reliable indicators of sexual dimorphism, offering crucial details for determining biological sex. The junction of the ramus and the mandibular body forms the gonial angle. The angle stands for angular measures that can be obtained using a range of imaging methods, including cone-beam computed tomography (CBCT), computed tomography (CT) scans, and cephalometric radiography. Quantifying these angles can also be done using conventional anthropometric techniques [10].

When analyzing cephalometric x-rays, the gonial angle is a crucial measure for figuring out an individual's growth pattern, class II patient mandibular rotation and extraction pattern evaluation, class III skeletal base patient surgical decision-making, and forensic medicine age estimation. An orthopantomogram can be used to simply calculate the left and right sides' gonial angles separately. The aim and objective are to analyze and evaluate the accuracy of gonial angles. To determine the age and gender of the population using the gonial angle.

## Materials and methods

G-Power software (version 3.1.9.4, Düsseldorf, Germany) was used to calculate the sample size to ensure a statistical power of 95% with a significance level (alpha error probability) of 0.05. The calculated sample size was 486, and we included a total of 500 samples to ensure adequate statistical power. Then the study was carried out using randomly chosen 1000 samples (500 males and 500 females) with an age range of 51 to 60 years and 61 to 70 years old. The obtained radiographs were preserved in the Department of Oral Medicine and Radiology, Saveetha Dental College and Hospitals, Chennai. The study was permitted by the Institutional Human Ethics Committee of Saveetha Dental College (IHEC/SDC/UG-2288/24/FORENSIC/078). The statistical analysis was performed using SPSS Statistics version 16.0 (SPSS Inc., Released 2007, SPSS for Windows, Version 16.0, Chicago, SPSS Inc.). The analysis included an accuracy test, analysis of variance (ANOVA), multiple regression, and discriminant analysis for gender on individual data.

Inclusion criteria

Instances where the sex is known and the accessibility of radiographs from orthopantomograms for every sample with head alignment that was contrasting and clearly visible were included in the study.

Exclusion criteria

Radiographs showing pathological lesions and radiographs, along with other artifacts making the gonial angle measurement difficult, were not included in the study.

Method of orthopantomogram measurement

**Figure 1:**
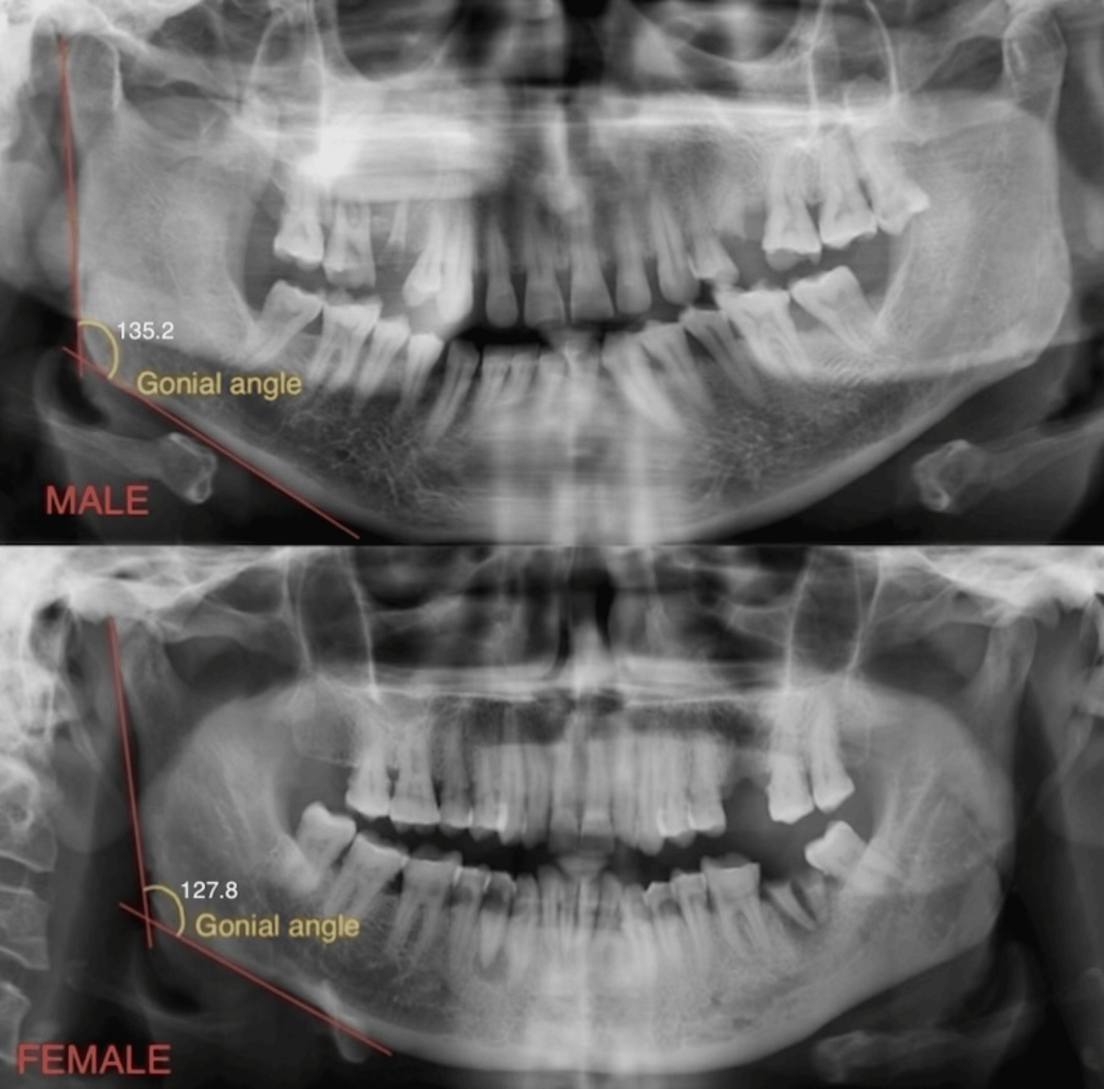
Representation of gonial angle in male and female (51 to 60 years) orthopantomograms, which measures from calculating the tangents to the mandible's lower border and the ramus' posterior border on the orthopantomogram.

The tangent line to the mandible's lower border and a line tangent to the ascending ramus and condyle's distal border were drawn on both sides to determine the gonial angle. Orthopantomograms (OPGs) were used to determine the age and gender of an individual aged 51 to 60 years; in males, it was measured at 135.2°, and in females, it was measured at 127.8°, as shown in Figure 1.

**Figure 2:**
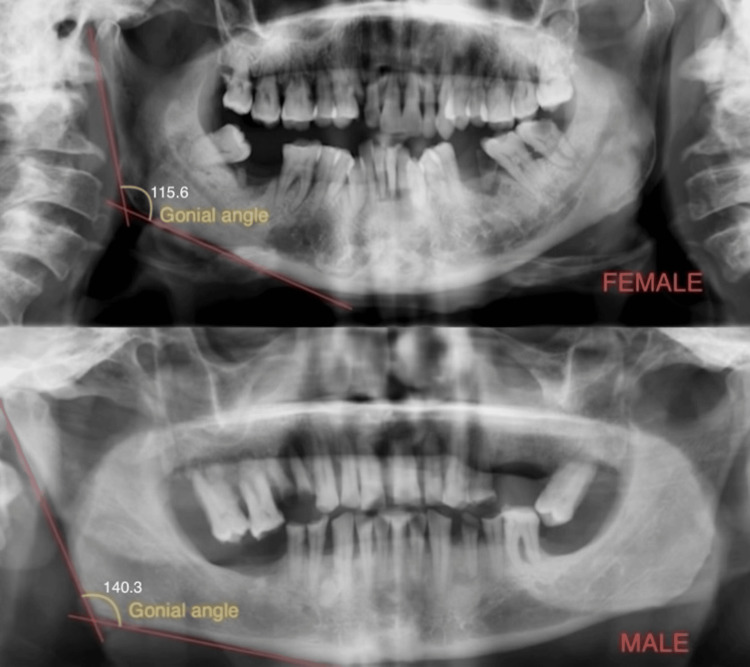
Representation of gonial angle in male and female (61 to 70 years) orthopantomogram.

Orthopantomograms (OPGs) were used to determine the age and gender of an individual aged 61 to 70 years; in females, it was measured at 115.6°, and in males, it was measured at 140.3°, as shown in Figure 2.

## Results

The current study involved two age groups of male and female samples, and in this study, random sampling was done to ensure a diverse and representative sample. The study included 1000 orthopantomograms, which were divided into two age groups (51-60 years and 61-70 years), with male 250 in numbers and female 250 in numbers in each group. To ensure the findings would be widely applicable for this present study, they are shown in Table 1.

**Table 1:** Distribution of sample size of male and female in the age group (51 to 60 years and 61 to 70 years).

Groups with Age Group	Male	Female	Total
Group I (51 to 60 years)	250	250	500
Group II (61 to 70 years)	250	250	500

The results were analyzed for both age groups, male and female; the gonial angle clearly decreases with age in this study, but there is no discernible pattern in the intergroup comparison. We also observed significant differences between the two groups. Males have a higher mean gonial angle (124.7370 degrees) compared to females (119.6371 degrees). The standard error is lower in females (0.20844) than in males (0.60998), suggesting more precise mean estimates in the female group. The p-value of <0.001 suggests that the disparity between males and females' mean gonial angles is statistically significant. The table indicates that there is a statistically significant difference in the gonial angle between males and females aged 51 to 60 years, with males having a larger gonial angle on average and females having a higher mean gonial angle (128.4322 degrees) compared to males (124.0529 degrees). The standard error is lower in males (0.14968) than in females (0.30028), suggesting more precise mean estimates in the male group. The p-value of <0.001 indicates that the disparity in mean gonial angles between males and females is statistically significant. The table indicates that there is a statistically significant difference in the gonial angle between males and females aged 61 to 70 years, with females having a larger gonial angle on average, which is shown in Table 2.

**Table 2:** Presents the descriptive statistics of the gonial angle (GA) in degrees for males and females aged 51 to 60 years and 61 to 70 years, including a comparison between the two groups.

Group Statistics													p-value	95% Confidence Interval of the Difference	
Mandibular Parameters			Age Group			Gender			Number (N)	Mean	Std. Deviation	Std. Error Mean		Lower	Upper
Gonial angle (GA) (degree)				51 - 60 yrs			Female		250	119.6371	3.29571	0.20844	≤0.001	-6.36631	-3.83332
							Male		250	124.7370	9.64468	0.60998	≤0.001	-6.36825	-3.83139
				61- 70 yrs			Male		250	128.4322	4.74790	0.30028	≤0.001	3.72010	5.03852
							Female		250	124.0529	2.36663	0.14968	≤0.001	3.71952	5.03910

The high p-value (0.700) and low t-value (0.385) indicate that variations in the gonial angle do not have a significant effect on the dependent variable. The formula couldn’t be derived as the P value is not significant, which is mentioned in Table 3.

**Table 3:** The gonial angle shows the significance of age range 51-60 years and 61-70 years. For the age group 51 to 60 years, a p-value of 0.584 is much greater than the typical threshold of 0.05, suggesting that the relationship between the gonial angle and the dependent variable is not statistically significant. The multiple regression analysis was done to identify the age and gender of this particular group, and the new formula derived from this current study was age = 58.384 + 0.10 x GA. For the age group of 61 to 70 years, the p-value of 0.700 is much greater than the typical threshold of 0.05, suggesting that the relationship between the gonial angle and the dependent variable is not statistically significant.

Mandibular Parameter	Age Group	Unstandardized Coefficient		Standardized Coefficient	t -value	p -value
		B	Std. error	Beta		
Constant (58.384)	51 - 60 years	0.10	0.18	0.026	0.548	0.584
Gonial angle (GA) (degree)						
Constant (68.815)	61 - 70 years	0.012	0.032	0.019	0.385	0.700
Gonial angle (GA) (degree)						

The initial group of cases had 96.8% of them correctly classified. The grouping of each gender was segregated for the age group 51 to 60 years, and both female and male groups were analyzed for gender prediction within the population. The results demonstrated nearly 96.8% accuracy in identifying both male and female genders. Additionally, 90.8% of the first instances that were grouped were appropriately classified. The grouping of each gender was segregated for the age group 61 to 70 years, and both female and male groups were analyzed for gender prediction within the population, as represented in Table 4.

**Table 4:** Prediction analysis of gender using gonial angle on age group 51 to 60 years and age group 61 to 70 years. The table details the precision of predicting age groups and genders. In the 51-60 age group, males were correctly identified as males with 99.2% accuracy and females with 94.4% accuracy. For the 61-70 age group, males were predicted with 93.6% accuracy, while females were predicted with 88% accuracy. Generally, males showed higher accuracy in being correctly identified by gender compared to females across both age groups.

Gender (N)	Age Group	Predicted Group Membership		Total
		Male (%)	Female (%)	
Male (N)	51 - 60 years	248 (99.2%)	2 (0.8%)	250
Female (N)		14 (5.6%)	236 (94.4%)	250
Male (N)	61 -70 years	234 (93.6%)	16 (6.4%)	250
Female (N)		31(12%)	219 (88%)	250

## Discussion

Gender estimation from human skeletal remains is a fundamental aspect of forensic anthropology. It relies on several anatomical features that exhibit sexual dimorphism. A mixture of features results in a more precise estimation of gender, while a single skeleton piece cannot establish gender with confidence [10]. Because mandibles have a high degree of sexual dimorphic features and are largely intact, they are employed for measurements [11]. Mandibular parameters are a set of characteristics that can be used to determine a person's biological sex. This method utilizes a number of mandibular morphological traits, such as the mandibular body dimensions, ramus height, and gonial angle [7]. Males and females grow mandibles at varying rates, which makes it possible to distinguish between the sexes [12]. Measurements and morphometry-based gender identification are reliable and useful for determining sex from the skull [13]. Identification of victims of disasters is considered to begin with determining the gender of the victim based on human skeletal remains [14].

In this current study, males aged 51 to 60 years have a higher mean gonial angle (124.7370 degrees) compared to females (119.6371 degrees). The standard error is lower in females (0.20844) than in males (0.60998), indicating more precise mean estimates in the female group. A P-value of less than 0.001 indicates a statistically significant variation in mean gonial angles among genders, with males having a larger gonial angle. In the 61- to 70-year-old age group, females have a higher mean gonial angle (128.4322 degrees) compared to males (124.0529 degrees). Here, the standard error is lower in males (0.14968) than in females (0.30028), suggesting more precise mean estimates in the male group. Again, a statistically significant difference is shown by a p-value of less than 0.001, with females having a larger gonial angle. Gender classification analysis for the 51- to 60-year-old age group showed 96.8% accuracy in identifying genders. For the 61- to 70-year-old age group, the gender prediction accuracy was 90.8% as well.

The Disaster Victim Identification Guide (2018), published by the International Criminal Police Organization (INTERPOL), states that antemortem and postmortem data are compared using primary methods (such as fingerprint, dental, and genetic tests) as well as auxiliary techniques (such as identification, anthropology, tattoos, and other topics) for human identification. Dental analysis is one of the most important primary approaches, since teeth can withstand extremely high temperatures and can also use DNA as a source of identification in emergency situations. Among the secondary approaches, forensic anthropology is unique. It is imperative that forensic anthropologists possess extensive training in the field of osteology to effectively handle the recognition of skeletal remains, notwithstanding their state of decomposition, burning, dismemberment, or skeletonization [15].

Anthropology, also known as "the science of humanity," studies all aspects of human life, including human biology and evolutionary background, as well as the social and cultural components that distinguish humans from other animal species. Numerous fields of research, encompassing linguistic, cultural, psychological, physical, social, and archaeology, are involved in this study of humans. The application of skeletal remains through postcranial anatomical features, facial approximation, frontal sinus variations, and unique cranial remains is a noteworthy aspect of the forensic anthropologist's job [1].

The importance, techniques, and developments in the use of gonial angles for anthropometric age and gender evaluation are examined in this paper. Anthropometry is very useful in forensic settings where skeletal remains are often valuable and delicate because it is a non-destructive procedure that maintains skeletal integrity [7].

The gonial angle is a crucial craniofacial characteristic that reveals details regarding the vertical dimensions and the facial bone’s symmetry. Both lateral cephalograms and panoramic radiography can be used to measure it [16]. The gonial angle can be used to anticipate the pattern of facial growth and is a measure of the steepness of the mandibular plan. This angle arises from the intersection of two lines: one line meets the mandibular inferior border, and the other line is tangent to the ramus and condyle, indicating the shape of the mandible based on the body's relationship to the ramus. When conducting near-age assessments in extreme circumstances such as mass disasters, excavated human remains, violent mutilations, missing persons, etc., the gonial angle can also be a useful tool [8,7]. 

According to Revant H et al., in order to investigate gonial angle, antegonial depth, and antegonial angle in connection to gender, age group, and dental status, the study evaluated 1060 panoramic radiographs. There was a discernible gender difference in these measurements, with males showing larger antegonial depths and smaller antegonial angles than females, even though there was no significant link between these measurements and age. Furthermore, notable distinctions were noted between the mandible's right and left sides. These results imply that, while these measurements are not appropriate for age assessment, they may be useful as forensic instruments for determining gender [17]. Compared to our study, the sample size was nearly equal, and the gonial angle measurements showed a significant link between age and gender.

The geometric and perpendicular dimensions of craniofacial characteristics can be measured using an orthopantomogram [7]. According to Ayesha H et al., this study compares age estimation methods using mandibular third molar development on panoramic radiographs (OPG) with mandibular linear dimensions on lateral cephalograms. Two hundred subjects (100 males and 100 females), aged nine to 20 years, underwent radiographic imaging. Linear measurements were taken from lateral cephalograms, while third-molar development was assessed on OPGs. The accuracy of age estimation was found to be 93.8% with OPG and 79.7% with lateral cephalograms, indicating that OPG analysis is more reliable than cephalometric parameters [18]. In our study, the age group of 51 to 60 years old had a 96.8% accuracy rate in gender identification according to the gender classification study. The gender prediction accuracy was 90.8% for the age range of 61 to 70 years old as well.

Limitations

Like any other technique for predicting age and gender, using mandibular features like the gonial angle alone has its limits. The limitations could make it more difficult to evaluate or extrapolate conclusions about the anatomy or function of the mandible. To address and reduce these variations in future analyses or interpretations, significant thought and possibly further research are required.

## Conclusions

According to this present study, gonial angle plays an important role in identifying age and gender and shows a 96.8% accuracy rate in gender identification according to a gender classification study in 51 to 60 years. The gender prediction accuracy was 90.8% for the age range of 61 to 70 years old as well. When compared to a known population norm, the evaluation of mandibular morphology using radiographic measurements may be helpful in determining an individual's age and gender.

## References

[1] Jayakrishnan JM, Reddy J, Vinod Kumar RB (2021). Role of forensic odontology and anthropology in the identification of human remains. J Oral Maxillofac Pathol.

[2] Ulusoy AT, Ozkara E (2022). Radiographic evaluation of the mandible to predict age and sex in subadults. Acta Odontol Scand.

[3] Sengupta N, Sarode SC, Sarode GS, Gadbail AR, Gondivkar S, Patil S, Patil S (2020). Analysis of 100 most cited articles on forensic odontology. Saudi Dent J.

[4] Khanagar SB, Vishwanathaiah S, Naik S (2021). Application and performance of artificial intelligence technology in forensic odontology - a systematic review. Leg Med (Tokyo).

[5] Murali K, Nirmal RM, Balakrishnan S, Shanmugam S, Altaf SK, Nandhini D (2023). Age estimation using cephalometrics- a cross-sectional study among teenagers of Salem district, Tamil Nadu. J Pharm Bioallied Sci.

[6] Dietrichkeit Pereira JG, Lima KF, Alves da Silva RH (2020). Mandibular measurements for sex and age estimation in Brazilian sampling. Acta Stomatol Croat.

[7] Arthanari A, Senthilkumar A, Ramalingam K, Prathap L, Ravindran V (2024). Exploring age and gender identification through mandibular parameters using orthopantomography: an observational study. Cureus.

[8] Upadhyay RB, Upadhyay J, Agrawal P, Rao NN (2012). Analysis of gonial angle in relation to age, gender, and dentition status by radiological and anthropometric methods. J Forensic Dent Sci.

[9] Arthanari A, Sureshbabu S, Ramalingam K, Ravindran V, Prathap L, Sitaraman P (2024). Analyzing mandibular characteristics for age and gender variation through digital radiographic techniques: a retrospective. Cureus.

[10] Arthanari A, Sureshbabu S, Ramalingam K, Prathap L, Ravindran V (2024). Forensic gender prediction by using mandibular morphometric indices: a panoramic radiograph study. Cureus.

[11] Okkesim A, Sezen Erhamza T (2020). Assessment of mandibular ramus for sex determination: retrospective study. J Oral Biol Craniofac Res.

[12] Shree B, Soni S, Sharma SK, Handge K, Kumar A, Das SS, Puri N (2023). Analytical study of mandible: prerequisite for sex determination. J Pharm Bioallied Sci.

[13] Ingaleshwar P, Bhosale S, Nimbulkar G, Britto F, Chandrappa PR, Hosur MB (2023). Mandibular ramus- an indicator for gender determination: a digital panoramic study in Bagalkot population. J Oral Maxillofac Pathol.

[14] Forrest A (2019). Forensic odontology in DVI: current practice and recent advances. Forensic Sci Res.

[15] Mello-Gentil T, Souza-Mello V (2022). Contributions of anatomy to forensic sex estimation: focus on head and neck bones. Forensic Sci Res.

[16] Tashkandi NE, Alnaqa NH, Al-Saif NM, Allam E (2024). Accuracy of gonial angle measurements using panoramic imaging versus Lateral Cephalograms in adults with different mandibular divergence patterns. J Multidiscip Healthc.

[17] Chole RH, Patil RN, Balsaraf Chole S, Gondivkar S, Gadbail AR, Yuwanati MB (2013). Association of mandible anatomy with age, gender, and dental status: a radiographic study. ISRN Radiol.

[18] Ayesha H, Zakaullah S, Ara SA, Priyanka A, Fatima A (2022). Age estimation using panoramic radiography and lateral cephalogram-A comparative study. Indian J Dent Res.

